# 7q21.3 Deletion involving enhancer sequences within the gene *DYNC1I1* presents with intellectual disability and split hand-split foot malformation with decreased penetrance

**DOI:** 10.1186/s13039-015-0139-2

**Published:** 2015-06-13

**Authors:** Sara Delgado, Milen Velinov

**Affiliations:** Bronx-Lebanon Hospital Center, New York, Bronx; Albert Einstein College of Medicine, New York, Bronx; Department of Human Genetics, New York State Institute for Basic Research in Developmental Disabilities, 1050 Forest Hill Rd, Staten Island, 10314 New York

**Keywords:** Deletion 7q21.3, Ectrodactyly, SHFM1l, *DYNC1I1*, *DLX5*, *DLX6*, *DSS1*, Gene enhancer

## Abstract

Split hand-split food malformation (SHFM) is a congenital defect of limb development that involves the central rays of the autopod and presents with median clefts of the hands and feet. It often includes syndactyly and aplasia/hypoplasia of the phalanges. SHFM is a genetic condition with high genetic heterogeneity, with at least 6 associated chromosomal loci. A locus in chromosomal region 7q21.3, associated with SHFM is referred to as SHFM1. Genes considered to be associated with SHFM1 are *DLX5* and *DLX6*. These two genes participate in the Wnt pathway that has a role in limb development. The gene *DYNC1I1,* located proximally (centromeric) to the SHFM1 locus was recently reported to include enhancer sequences involved in limb development in its exons 15 and 17. These sequences were shown to cis-regulate the function of the adjacent SHFM associated genes. We report a family, in which the father and three of his sons carry an approximately 1 Mb deletion in this chromosomal region, arr[hg19]7q21.3(94,769,383-95,801,045)x1. The deleted region is located proximally (centromerically) adjacent to the SHFM region at 7q21.3. It does not include the SHFM candidate genes *DLX5* and *DLX6*, but includes the enhancer sequences within *DYNC111* and six other genes centromeric to *DYNC1I1*. All deletion carriers have various degrees of intellectual disability while two of them have SHFM. This family is the eighth reported family where a chromosome 7q21.3 deletion co-segregating with SHFM involves the enhancer regions within gene *DYNC111*, but does not involve the genes *DLX5* and *DLX 6.* This is also the third family where decreased penetrance of enhancer-associated SHFM is demonstrated. Intellectual disability was not observed in the previously reported families and may be associated with deficiency of one or more of the 6 genes included in the reported deletion centromeric to *DYNC1I1*.

## Background

Split hand-split foot malformation (SHFM), also referred to as ectrodactyly, is a group of genetic conditions affecting the limb formation. Limb defects associated with SHFM involve the central autopod and manifest as midline clefts of hands and/or feet. Six loci associated with SHFM were described to-date. Type 1 SHFM1 is associated with defects in chromosomal region 7q21.3 [1]. Deletions, duplications and complex chromosomal rearrangements of this chromosomal region, as well as point mutations in the gene *DLX5* located in the region were reported in individuals with SHFM1 [2–6]. Recently two enhancer sequences expressed in regions of limb development in mouse embryos were identified within the exons 15 and 17 of the gene *DYNC1I1* that maps centromeric to *DLX5/6*. These enhancer sequences were shown to regulate the expression of *DLX5/6* and thus have a role in embryonic limb development [7, 8]. Seven families with isolated or familial SHFM, who have deletions involving these enhancers but not the genes *DLX5/6* were reported to-date [9–12]. In two of these reports decreased penetrance of these chromosomal aberrations was demonstrated [9, 12]. In one of these reports Rattanasopha et al. also showed maternal imprinting of the genes DLX5 and 6 in osteoblasts [12]. We report an additional family where four relatives carry a 7q21.3 deletion involving *DYNC1I1* and 6 other proximally located genes. The carriers of this deletion have SHFM with reduced penetrance and intellectual disability.

### Case presentation

**Fig. 1:**
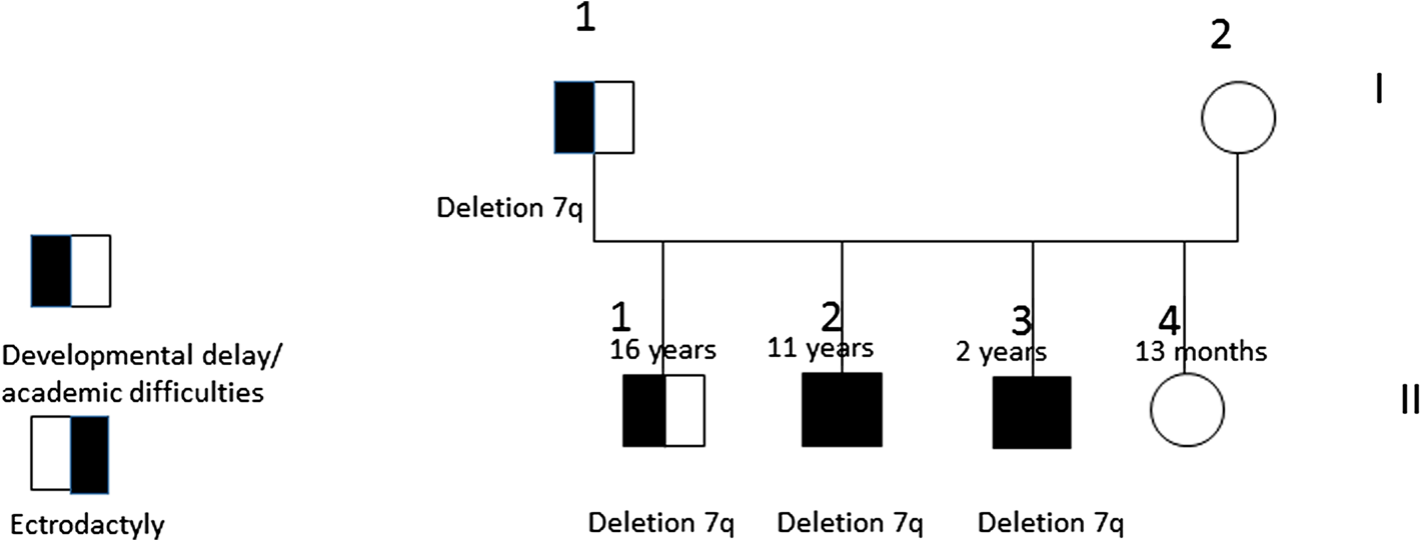
Pedigree of the reported family

The proband, individual II-2 on Fig. 1, was first evaluated at age 9 years. He was born premature at 34 weeks gestation with C-section because of breech presentation. His birth weight was 4 lbs 8 oz. He stayed in the nursery for one week due to his prematurity. At birth he was noted to have bilateral split foot, but his hands were unaffected. This boy had normal early development. At school he was noted to have significant academic difficulties and repeated first and second grade. He now attends special education class. On conversation he had fluent speech. He was toilet trained. On physical exam all his growth parameters were at the 50^th^ percentile for age. He did not have dysmorphic features. Both his feet had prominent midline defects (split foot) and severe syndactyly. His Cardiology exam and ECHO exam were normal. On his last psychology evaluation at age 11 years his overall functioning was determined to be at 6 years level. His score on ‘Children’s Global Assessment Scale” (CGAS) [13] was 55 in 1–100 scale, which is in the range described as: “Variable functioning with sporadic difficulties or symptoms in several but not all social areas”. He was unable to count after 11, was unable to name weekdays one after another and was unable to name some colors. He also had aggressive behavior while interacting with his classmates. In addition to his intellectual disability he was concluded to have adjustment disorder.

Oligo SNP array, Affimetrix Cytoscan HD (Quest Diagnostic, San Juan CA) identified an approximately 1 Mb deletion in chromosomal region 7q21.3. The reported coordinates of this abnormality were arr[hg19]7q21.3 (94,769,383-95,801,045). The chromosomal deletion included the genes *PPP1R9A, PON1, PON3, PON2, ASB4, PDK4, DYNC1I1* and *SLC25A13*. All siblings and both parents were studied using the same technology. The proband's brother, individual II-1, was first evaluated by us at age 13 years. This patient had uncomplicated perinatal period. He did not have split hand/foot, but his nails were mildly hypoplastic. He had significant speech delay. Reportedly he was unable to make sentences until he was 3 years old. He had academic difficulties in school and now attends a special education class. On his latest psychology evaluation at age 15 he had a CGAS score of 55 similarly to his younger brother. He was also found to have a mood disorder with periods of depression. His ECHO exam was normal.

**Fig. 2:**
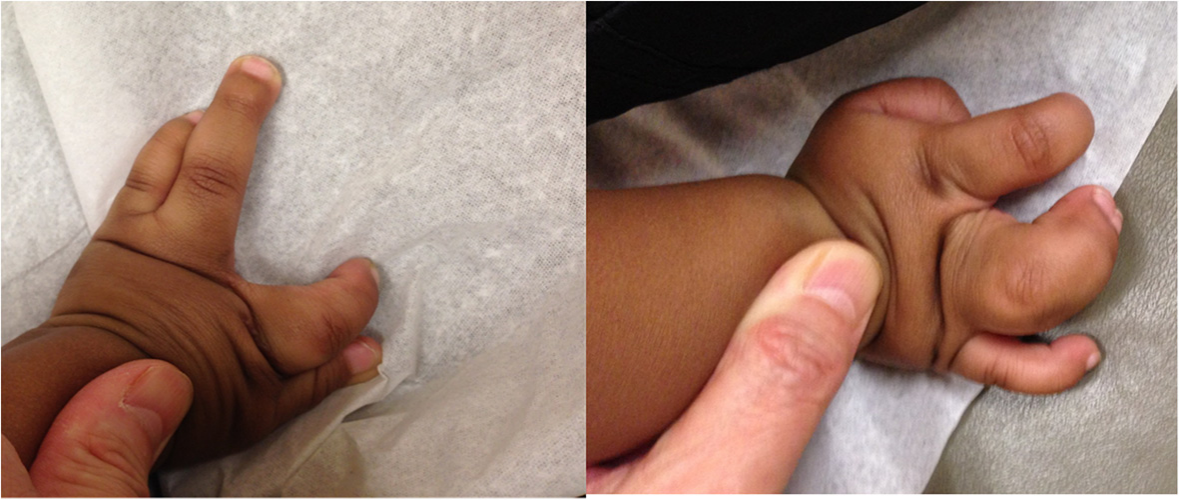
Bilateral split hand malformation observed in individual II-3

The proband’s second brother, individual II-3 was first evaluated at age 3 months. He was born after pregnancy complicated with gestational diabetes. He was born at full term. His birth weight was 8 lbs 3 oz. At birth he was noted to have split-hand malformation in both hands, but not on his feet (see Fig. 2). He had midline defects and severe finger syndactyly. His feet were unaffected except for unusual position of his toe nails that appeared detached of their beds and pointing upwards. This sibling's development was normal during his first year of life. He was noted to have developmental delay mainly affecting his speech at about 20 months of age. Audiology screening was normal. He was found to have ankyloglossia and had frenulectomy at 20 months. His ECHO exam was normal.

These three male siblings and their father were positive for the 7q21.3 deletion (see Fig. 1). The father did not have ectrodactyly and reportedly had mild learning disability while in school.

## Conclusions

**Fig. 3:**
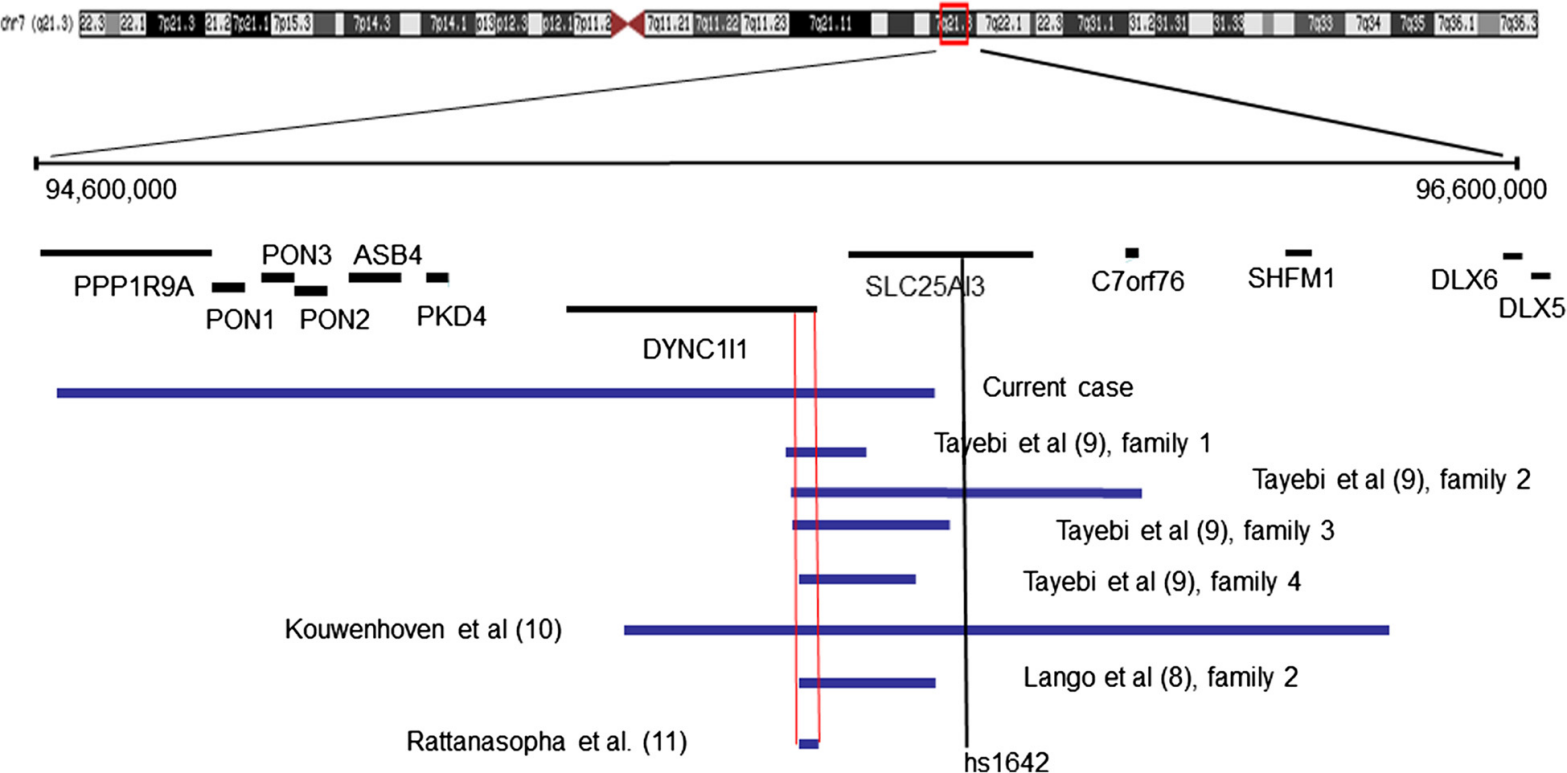
The SHFM1 chromosomal region is shown. The previously reported deletions are shown in blue. The enhancer sequences in gene *DYNC1I1* are indicated with vertical red lines. The sequence hs1642 is indicated with vertical black line. References for the reports are shown in parentheses

Our family and seven previously reported families [9–12] constitute a distinct subtype of SHFM1 where the chromosomal abnormality do not include the traditionally SHFM- associated genes *DLX5/6*, but rather involve the associated cis-enhancer sequences within the adjacent gene *DYNC111.* (Fig. 3)*.* We refer to this subgroup as eSHFM1 (enhancer type I). This enhancer type SHFM shows decreased penetrance regarding the limb defects as it is seen in our family and in two of the previously reported families [9, 12]. All affected individuals in our family had inherited their deletion form their father which is in accordance with the findings of Rattanasopha et al. regarding maternal imprinting [12]. Among the previously reported larger families with enhancer deletions only one affected individual had inherited his deletion from his unaffected mother [9]. This individual’s phenotype cannot be explained with maternal imprinting and additional studies may be needed in this family in order to address this discrepancy. The overall penetrance of limb deformity in such enhancer deletions considering all reported cases with known inheritance is around 40 %. At this time the molecular basis of the observed reduced penetrance is unknown*.* The maternal imprinting of *DLX5/6* shown by Rattanashopha et al. cannot explain this reduced penetrance because some of the unaffected individuals, including in our family, had inherited the 7q21.3 deletions from their fathers. Due to the reported maternal imprinting of *DLX5/6* the inheritance of enhancer deletion from one’s mother may be associated with lower risk for SHFM. Our family did not have hearing deficit as it was observed in some of the other reported families with eSHFM1. It seems that hearing deficit due to SHFM1 related chromosomal deletions is associated with involvement of a more telomerically (distally) located sequence in this area. The previously reported families with hearing loss have deletions that include the brain enhancer element hs 1642 [10] that is located within gene *SLC25A13*. Our family corroborates the suggestion of Taybi et al. [10] that deletion of hs1642 is associated with hearing loss since the affected members in our family do not have hearing loss and the hs 1642 sequence is not involved in their deletion (Fig. 3). The apparently mild intellectual disability observed in our family is not present in the other reported families and seem to be associated with hemozygocity for a gene located proximally to *DYNC111* (Fig. 3). One strong candidate gene for such manifestations is the gene *PPP1R9A*. This gene was shown to be expressed in the brain and to participate in neuronal development [14].

### Consent

Consent for publishing the current report and the medical photography was obtained from the family.
